# High heritability of coral calcification rates and evolutionary potential under ocean acidification

**DOI:** 10.1038/s41598-019-56313-1

**Published:** 2019-12-31

**Authors:** Christopher P. Jury, Mia N. Delano, Robert J. Toonen

**Affiliations:** 1 Hawai‘i Institute of Marine Biology, School of Ocean & Earth Sciences & Technology, University of Hawai‘i at Mānoa, P.O. Box 1346, Kāne‘ohe, HI 96744 USA; 2 Global Environmental Science, School of Ocean & Earth Sciences & Technology, University of Hawai‘i at Mānoa, Honolulu, HI USA

**Keywords:** Climate-change ecology, Evolutionary ecology, Tropical ecology

## Abstract

Estimates of heritability inform evolutionary potential and the likely outcome of many management actions, but such estimates remain scarce for marine organisms. Here, we report high heritability of calcification rate among the eight most dominant Hawaiian coral species under reduced pH simulating future ocean conditions. Coral colonies were sampled from up to six locations across a natural mosaic in seawater chemistry throughout Hawaiʻi and fragmented into clonal replicates maintained under both ambient and high pCO_2_ conditions. Broad sense heritability of calcification rates was high among all eight species, ranging from a low of 0.32 in *Porites evermanni* to a high of 0.61 in *Porites compressa*. The overall results were inconsistent with short-term acclimatization to the local environment or adaptation to the mean or ideal conditions. Similarly, in ‘local vs. foreign’ and ‘home vs. away’ tests there was no clear signature of local adaptation. Instead, the data are most consistent with a protected polymorphism as the mechanism which maintains differential pH tolerance within the populations. Substantial individual variation, coupled with high heritability and large population sizes, imply considerable scope for natural selection and adaptive capacity, which has major implications for evolutionary potential and management of corals in response to climate change.

## Introduction

Coral reefs are home to roughly one-quarter of marine biodiversity, provide food for hundreds of millions of people, and billions of dollars annually worth of ecosystem services, but reefs are increasingly threatened by global change and local human impacts^1,2^. Even under aggressive mitigation scenarios, coral reefs are predicted to suffer catastrophic global losses over the coming decades due to climate change stressors^3–5^. Such predictions, and many management actions being proposed to save coral reefs^6^, are based on the widely held assumption that scleractinian corals have low evolutionary potential and cannot adapt to anthropogenic global change over relevant timescales^1,7,8^. However, recent data suggest thermal tolerance can increase within decades in natural populations^9,10^, and models indicate that if corals possess even a moderate ability for acclimation or adaptation to changing climate, then the future of coral reefs may not be quite so bleak^11,12^. Despite the critical importance of adaptive potential, and the fundamental role it plays in the outcome of such proposed management actions as assisted evolution^13^, the heritability of temperature and pH tolerance as well as the spatial and temporal capacity for corals to adapt to novel environmental conditions remains almost entirely unknown^14^.

Some corals can survive complete skeletal dissolution in the laboratory^15^ and these results have been interpreted as support for the ‘naked coral hypothesis’^16^ wherein the loss of skeletons would become favored by selection during periods of inhospitable seawater chemistry (but see Jury and Jokiel^17^ for counterarguments to this hypothesis). However, at CO_2_ vent sites in the Mediterranean and in the Pacific^18,19^ corals decline in abundance and eventually disappear as pH decreases, suggesting that reduced calcification under acidification does indeed come at a cost to fitness. The question of whether corals are capable of adaptation to ocean acidification relies critically on whether pH tolerance in scleractinian corals is a heritable trait with individual variation upon which natural selection can act. In the absence of heritable individual variation, there can be no adaptation, but despite widespread concern and an extensive literature on the impact of ocean acidification on corals, the heritability of calcification rates under reduced pH remains unknown and unmeasured in scleractinians to date. Multiple recent studies demonstrate that corals exhibit seemingly adaptive responses to locally warmer or more acidic habitats^19–24^. It is well-known that different species of coral have different pH and temperature tolerances and react differently to experimental acidification^19,25,26^. In Hawaiʻi, three species of coral (*Montipora capitata*, *Pocillopora acuta*, and *Porites compressa*) thrive under naturally warmer and more acidic conditions within Kāneʻohe Bay, Oʻahu, relative to colonies of the same species located elsewhere around the island^10,12,27–30^. Significant differences in responses among conspecific individuals from different areas within a natural environmental mosaic suggests that there may be variation among individuals that results from either physiological compensation to reduced pH (acclimatization) or natural selection against individuals that are most sensitive to the naturally more acidic waters of Kāneʻohe Bay^10,27^. If the variation among individuals collected from different locations is a result of acclimatization, bringing corals from the different locations into a common garden for an extended period should result in changes to pH sensitivity through time. If, on the other hand, selection removes individuals that cannot tolerate reduced pH, we should see the persistence of individual variation during common garden experiments and be able to estimate the heritable genetic component of pH tolerance. These mechanisms are not mutually exclusive, and each could potentially contribute to the observed variation in acidification tolerance.

Here we examine the heritable component of pH tolerances for the eight most common reef-building coral species in Hawaiʻi that collectively comprise >90% of coral cover in the State^31,32^. We estimate *broad-sense heritability* (H^2^), which includes genetic, maternal, epigenetic, and other heritable sources of variation that together control the sensitivity of a population to reduced pH (i.e., the proportion of phenotypic variation in calcification rate that can be explained by additive genetic variance, as well as by non-additive genetic factors such as epistasis). Phenotype is the result of both heritable and environmental influences, but only heritable variation can be passed to offspring and acted upon by natural selection; thus, the heritable component of phenotypic expression governs the potential for a population to adapt under selective pressure. For coral reefs to be capable of adaptation to future climate change, there must be heritable genetic variation for fitness-related traits within populations on which selection may act^33^. Previous work has shown such heritable differences for thermal tolerance in corals^34^, but to date whether differences in tolerance for pH stress are heritable and subject to selection remains unknown. Here we examine variation among individual colonies from a natural pH mosaic around the island of Oʻahu, Hawaiʻi and, using clonal nubbins (ramets) in a virtual ‘reciprocal transplant’ mesocosm experiment, estimate the heritability of variation among colonies in their calcification rate under experimentally increased CO_2_ levels. Such information provides an important first step toward understanding the evolutionary potential for corals to adapt to ocean acidification.

## Materials and Methods

### Sample collection

**Figure 1:**
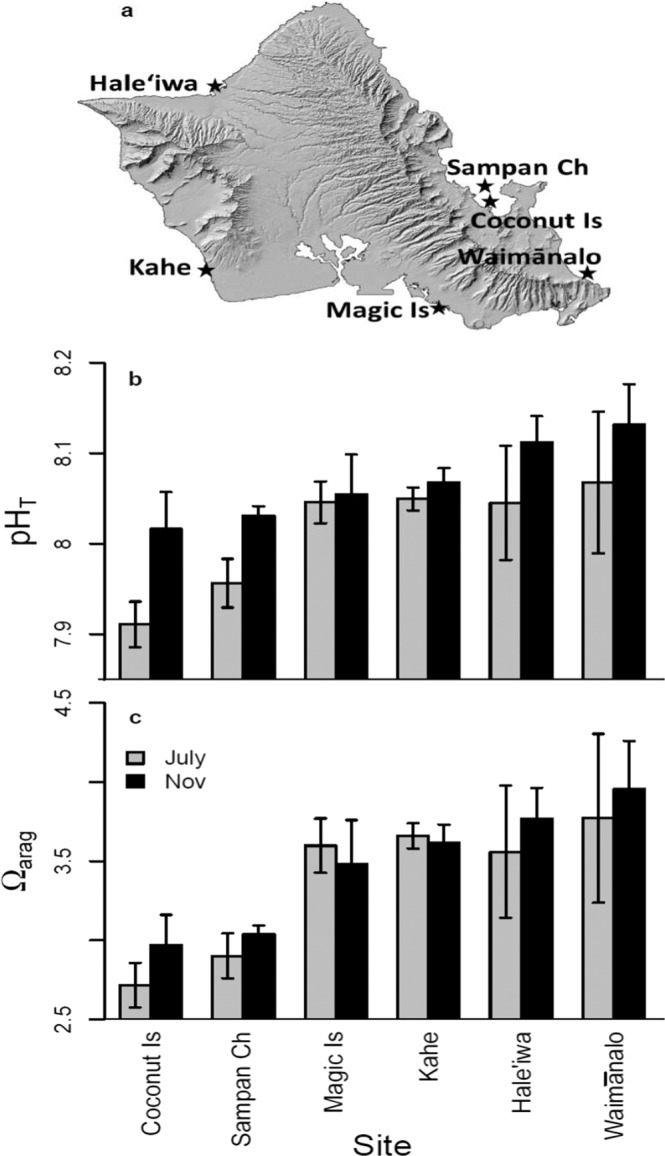
Six collection locations for corals around the island of Oʻahu, HI, USA as indicated by stars (**a**) as well as *in situ* daytime pH (**b**) and aragonite saturation state (**c**) at each site from water samples taken in July and November, 2016. Bottle samples were collected at 0800, 1200, and 1600 hr. Data shown as mean ± SEM of the three daytime samples. Coral species collected at each site are described in Table 1.

Samples of each of the most common reef-building corals in Hawaiʻi (*Pocillopora acuta*, *P. meandrina*, *Montipora capitata*, *M. patula*, *M. flabellata*, *Porites compressa*, *P. evermanni*, and *P. lobata*) were collected from six locations around the island of Oʻahu, Hawaiʻi (Table 1), spanning a natural mosaic in carbonate chemistry (Fig. 1). Species were only sampled at sites where colonies were relatively abundant, but every species was collected at a minimum of two sites with differing typical conditions (Fig. 1). Coral genets (genetically distinct colonies) were selected haphazardly at each site and conspecifics were separated by at least 5 m to minimize the chances of accidentally sampling clones or biasing the sampling toward particular microenvironments. While we did not genotype the corals in this study, prior work suggests a very low probability that any of the corals were clonally derived (<1% chance based on previous work^35–38^). Coral genets were sampled with a hammer and chisel on snorkel from a depth of 0.5–5 m and returned to the Hawaiʻi Institute of Marine Biology where they were allowed to recover. Six replicate clonal ramets (~3 cm nubbins) were cut from each sampled coral genet using a band saw prior to being mounted on a labeled plaster plug using cyanoacrylate gel. Experimental fragments were allowed to recover in flow-through aquaria for ~6 months to standardize the short-term history for all colonies prior to starting the experiment (Fig. S1).

**Table 1:** Collection locations (from Fig. 1) and number of coral genets (individual parent colonies) sampled at each site for each of the eight species of scleractinian coral collected for this study.

Species	Coconut Island	Sampan Channel	Magic Island	Kahe	Hale‘iwa	Waimānalo
*Pocillopora acuta*	4	2			2	
*P. meandrina*		3		4	4	4
*Montipora capitata*	3	3		3	3	3
*M. flablellata*		6			2	
*M. patula*		4		3	4	4
*Porites compressa*	3	3	3		3	3
*P. evermanni*		3	3	3	3	3
*P. lobata*		3	3	3	3	3

Sampling at each site was limited to species which occurred in relatively high density.

Population genetic structure has been characterized for only two of these species in Hawai‘i (*Montipora capitata*^37^ and *Porites lobata*^38^). Both these species show high levels of connectivity at the scale of individual islands, with the emergence of significant genetic structure over larger distances among islands. Likewise, both empirical^39^ and modeling studies^40,41^ indicate substantial scope for connectivity around O‘ahu at the scales considered here (~50 km). Hence, available evidence suggests that conspecifics from different sites are likely to be part of the same population, however, to be conservative we take unknown genetic structure among sites into account in our analyses, as described below.

### Mesocosm experiment

Six ramets from each of the 8–15 genets per species (depending on the number of sites at which each species was abundant) were then haphazardly divided into groups of 3, and the groups were randomly allocated to either a high or a low pH mesocosm. Four flow-through mesocosm tanks (300 L) were used for the experiment with two mesocosms randomly assigned to each pH level and a set of 3 ramets randomly assigned to each treatment (Fig. S1). This design was as close as was feasible to a reciprocal transplant experiment because the State of Hawaiʻi would not permit the transplant of living corals among locations. Each tank was covered by a 60% shade cloth to give the corals an average mid-day irradiance of ~800 μmol photon m^−2^ s^−1^, similar to conditions in their natural habitat. Each tank had constant raw seawater flow-through (~400 L hr^−1^) with a MaxiJet 1200 propeller circulation pump (~4900 L hr^−1^) for bulk water flow, and tanks were cleaned twice per week. Corals were exposed to the pH treatments for 6 weeks (which, based on our prior work, was long enough to generate precise growth estimates^10^) and their calcification rates were assessed using the buoyant weighing technique^42^.

### Chemistry monitoring

Tanks assigned to the low pH treatment were dosed with CO_2_ gas to achieve pH levels that were reduced by an average ~0.4 (to 7.6), while high pH tanks received ambient seawater with an average pH ~8.0 (Table 2). Twice weekly measurements of pH, alkalinity, temperature and salinity were taken from each tank at 1200 hr. Periodic spot checks throughout the diel cycle with a pH meter during the experiment revealed that nighttime pH was ~0.1–0.15 units lower than the daytime values. Temperature and salinity were measured with a YSI multimeter, total alkalinity was measured using an autotitrator, and pH was assessed spectrophotometrically with m-cresol purple, following standard protocols^43^. Additional chemistry parameters were calculated with CO2sys^44^.

**Table 2:** Carbonate chemistry (mean values ± standard errors) for each parameter measured in the experimental mesocosm aquariums.

Tank	pH_T_	Temperature (°C)	Alkalinity (μmol kg^−1^)	Salinity (psu)	pCO_2_ (μatm)	Ω_arag_
**1**	7.62 ± 0.02	25.6 ± 0.2	2142 ± 8	34.4 ± 0.1	1168 ± 55	1.41 ± 0.06
**2**	8.03 ± 0.01	25.6 ± 0.2	2142 ± 7	34.4 ± 0.1	391 ± 10	3.08 ± 0.06
**3**	7.65 ± 0.02	25.5 ± 0.2	2145 ± 7	34.4 ± 0.1	1093 ± 52	1.49 ± 0.06
**4**	8.04 ± 0.01	25.6 ± 0.2	2141 ± 8	34.4 ± 0.1	375 ± 11	3.17 ± 0.06

Water samples were collected twice per week at 1200 hr during the 6 week experiment (n = 13). Tanks 1 & 3 were randomly selected as the low pH treatment leaving 2 & 4 as the ambient pH treatment.

Carbonate chemistry is known for two of the collection sites^29,30^, but had not yet been characterized at the other four sites. Bottle samples were collected from each site at 0800, 1200, and 1600 hr during one day each in contrasting seasons (July and November, 2016). Sea water was collected near the center of each coral sampling area at a height of ~20 cm above the substrate (sampling depth of ~0.5–3 m) at each site. After collection, water samples were returned to the laboratory and analyzed for salinity, total alkalinity, and pH as above. *In situ* temperature was measured with a mercury thermometer calibrated against the YSI multimeter.

### Statistical analyses

A few coral nubbins (3% of the total, or 0–9% for each species) died before the end of the study (3 *Pocillopora acuta*, 4 *P. meandrina*, 1 *Montipora capitata*, 1 *M. flabellata*, 2 *M. patula*, 0 *Porites compressa*, 8 *P. evermanni*, 1 *P. lobata*). All analyses were performed both without and with these dead corals included (assuming a calcification rate of zero for dead nubbins) to assess whether their inclusion vs. exclusion biased the results. Coral calcification responses to the treatments were assessed using the repeatability function in the R package heritability^45^. This function estimates heritability based on the classical work of Falconer^46^ as further developed by Singh *et al*.^47^ and Lynch & Walsh^48^. heritability relies on an ANOVA to estimate the genotypic and environmental variance components of broad sense heritability defined as H^2^ = V_g_/(V_g_ + V_e_), where V_g_ is the genetic variance and (V_g_ + V_e_) is the phenotypic variance, based on the mean squared error (MSE) from the ANOVA. This method makes the same assumptions about the data as those intrinsic to ANOVA from which the heritability estimate is derived. Line repeatability was set to ‘false’ for heritability to estimate H^2^ from individual ramets, as opposed to pooling nubbins within each genotype. The package also estimates 95% confidence intervals for heritability^47^. For these analyses H^2^ was estimated with and without both pH and collection site as additional covariates to consider the effects of treatment or potential genetic structure among locations on the heritability estimates^47^.

To assess possible tank effects, an ANOVA was run on the pooled calcification data with pH as a fixed factor and tank as a nested factor. Tank effects were not significant (p = 0.358, data not shown), so this factor was dropped from the analysis. ANOVAs were then run to assess the effect of treatment pH, collection site, and parent colony as well as pH of origin on calcification rates of each species with each treated as a fixed factor, followed by a Tukey HSD *post hoc* test. To contrast acidification tolerance by pH of origin, linear mixed-effects models (LMM) were fit for each species using the package lme4^49^ with pH and pH of origin as fixed effects and parent colony and site as random effects, followed by an ANCOVA and pairwise comparisons using the lstrends and pairs functions, respectively, in the package lsmeans^50^. Likewise, to contrast acidification tolerance among species an LMM was fit with pH and species as fixed effects and parent colony and site as random effects, followed by an ANCOVA and pairwise comparisons. All analyses were performed with R v.3.5.2^51^.

## Results

### Environmental parameters

Conditions of flow, irradiance, temperature and water turn-over rate were all held similar among the four mesocosms. Carbonate chemistry was manipulated by direct CO_2_ gas addition as outlined above, with the average daytime pH levels over the course of the experiment being maintained at 7.62 ± 0.02 and 7.65 ± 0.02 in the two low pH treatment tanks, and pH levels at 8.03 ± 0.01 and 8.04 ± 0.01 in the two ambient pH tanks (Table 2). Results of the *in situ* chemistry sampling revealed that the pH and aragonite saturation state (Ω_arag_) were, on average, lower at the two Kāne‘ohe Bay sites (Coconut Island and Sampan Channel) as compared to the other four sites (Magic Island, Kahe, Hale‘iwa, and Waimānalo; Fig. 1), which is consistent with prior sampling^29,30^. We did not characterize the within-site chemistry variation in this study, but previous studies report variation in mean pH on the order of ± 0.02 units at our Coconut Island and Waimānalo sampling locations^29,30^, or about ± 15% of the difference in mean pH between these sites.

### Calcification rates

**Figure 2:**
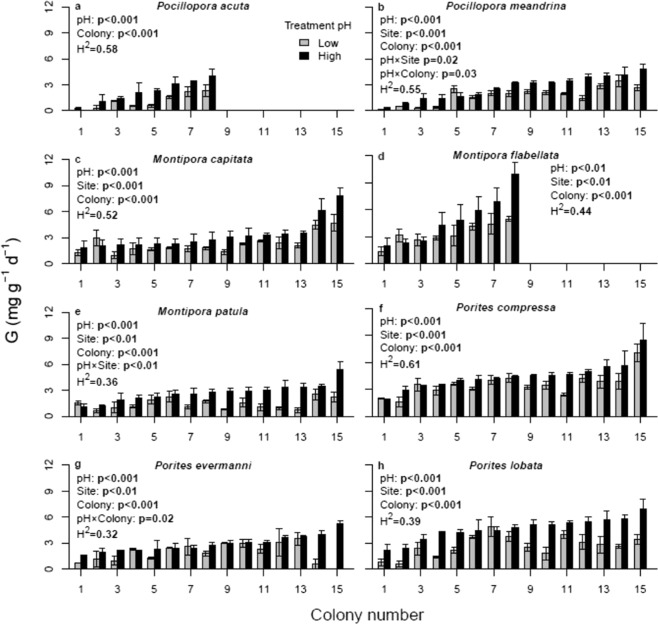
Mean calcification rate ( ± SEM) under ambient (black, high) and reduced (grey, low) pH conditions of replicate ramets (clonal coral nubbins) sampled from each of the 8–15 genets (individual colonies) of each species. Figure legends include significant ANOVA test results and broad-sense heritability estimates for each species from the experiments; p-values in bold are significant at α = 0.05.

**Figure 3:**
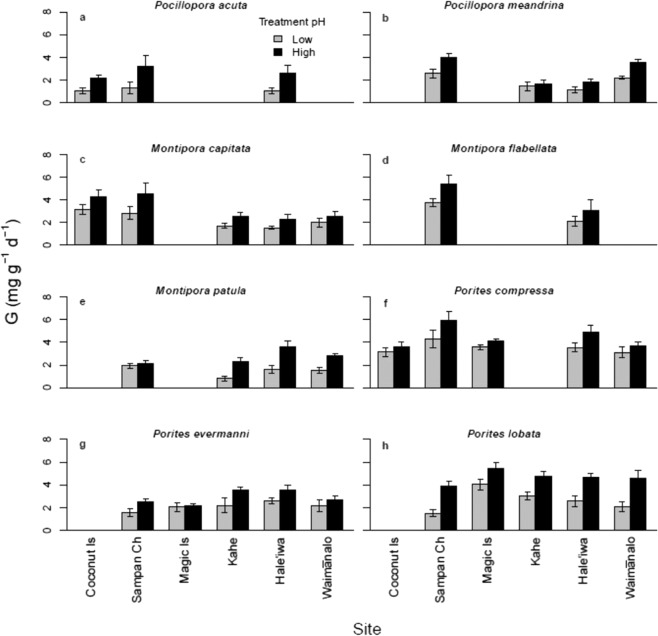
Mean calcification rate ( ± SEM) under ambient (black, high) and reduced (grey, low) pH conditions for coral genets collected from each location. See Fig. 2, Table S1 for ANOVA results.

Calcification varied significantly (p < 0.01) among both colony and pH treatments for each of the eight species (Fig. 2; Table S1). There were also significant non-interactive site effects for five of the species (*Montipora capitata*, *M. flabellata*, *Porites compressa*, *P. evermanni*, and *P. lobata*; Fig. 3; Table S1). For *Montipora patula* and *Pocillopora meandrina* there are also significant (p < 0.05) pH × Site interactions, indicating that some collection locations harbor a higher proportion of pH tolerant individuals than others, In *Montipora patula*, colonies from the low pH Sampan Channel site were significantly less sensitive to acidification than those from high pH Kahe, Hale‘iwa, or Waimānalo sites; in *Pocillopora meandrina*, colonies from the high pH Kahe and Hale‘iwa sites were significantly less sensitive to reduced pH than those from the low pH Sampan Channel site or the high pH Waimānalo site (Fig. 3; Table S1). Additionally, *Pocillopora meandrina* and *Porites evermanni* show significant (p < 0.05) pH × Colony interactions, indicating that individuals vary significantly in their response to experimental acidification within sites and that some coral colonies were significantly more sensitive to acidification than others from the same site. It is also noteworthy that among these species in which there is a significant interaction between pH × Site or pH × Colony, some individuals showed *increased* growth under acidification whereas others showed a marked decline in growth under reduced pH (e.g., compare *Montipora patula* colony 1 to 13, Fig. 2). For other species, such as *Porites lobata*, which do not have a significant pH × Colony interaction, the impact of increased CO_2_ on growth rate is highly variable and some individuals show the same positive impact of acidification (e.g., compare colony 7 to 4, Fig. 2), but the variation among individuals is not significant (p = 0.06), perhaps because of the relatively low sample size. These findings were essentially unaltered whether the dead nubbins were included or dropped from the analyses, likely due to the small number of affected corals.

### Heritability

Broad sense heritability (H^2^) estimates of calcification rates were uniformly high among all eight species of coral, ranging from 0.32 in *Porites evermanni* to 0.61 in *Porites compressa*. Confidence intervals for heritability were relatively large (Table 3), but typical of such estimates from previous work^34,47^. Analyses were repeated without treatment pH and collection site as covariates to assess if these factors contribute significantly to the heritability estimates. When pH was excluded as a covariate, heritability estimates were slightly lowered, whereas when site was excluded as a covariate, effects varied by species; none of the estimates differed significantly, however, regardless of the covariates included in the model (Table S2). These results indicate that calcification rates are strongly heritable in each of these species regardless of pH environment or assumptions about population genetic structure among collection sites, though treatment pH and collection location do contribute to the variance explained by the models. As with the ANOVAs, these estimates did not change significantly whether the dead nubbins were included or dropped from the analysis (Table S2).

**Table 3:** Broad-sense heritability **(**H^2^) values and the 95% confidence interval for the estimate of heritability of calcification rate for each of the eight Hawaiian coral species tested (all are significant, p < 0.05).

Species	H^2^	95% CI	Genotypic Variance	Environmental Variance	Effective # Replicates
*Pocillopora acuta*	0.58	0.29–0.87	0.975	0.717	5.61
*P. meandrina*	0.55	0.33–0.77	0.629	0.521	5.73
*Montipora capitata*	0.52	0.31–0.76	1.341	1.219	5.93
*M. patula*	0.36	0.16–0.63	0.435	0.776	5.86
*M. flabellata*	0.44	0.16–0.80	2.742	3.497	5.87
*Porites compressa*	0.61	0.41–0.81	1.684	1.069	6
*P. evermanni*	0.32	0.11–0.60	0.443	0.959	5.46
*P. lobata*	0.39	0.18–0.65	0.826	1.319	5.93

Treatment pH and collection location were set as covariates (see Table S2 for a comparison of results using other combinations of covariates). Additional columns correspond to estimates of genotypic variance, environmental variance, and the effective number of replicates from ANOVA results as outlined in the text.

### Tests for local adaptation under ‘home vs. away’ and ‘local vs. foreign’ conditions

**Figure 4:**
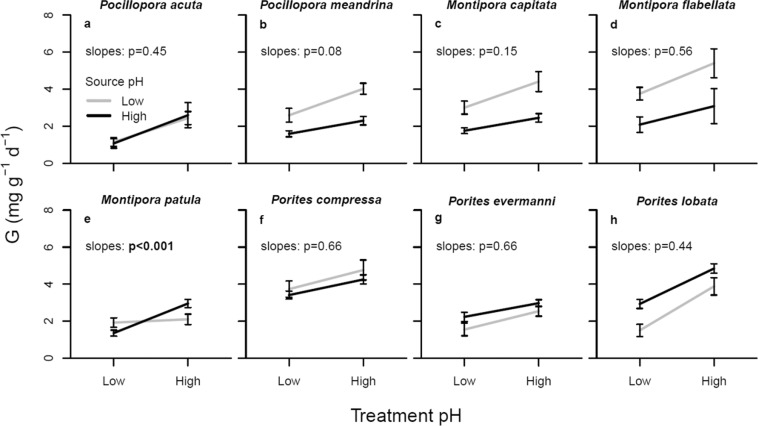
Mean calcification rate ( ± SEM) of coral ramets (clonal nubbins) of each of the eight primary reef building coral species in the Hawaiian Archipelago, collected from naturally high (black) or low (grey) pH sites around Oʻahu (Fig. 1, Table 1), when held under either high or reduced pH conditions (local vs. foreign and home vs. away conditions) in the laboratory. Legend includes ANCOVA results for contrasts of slopes by source pH; only *M. patula* shows a signficant difference in pH tolerance according to source pH (p < 0.05). See Table S3 for additional ANOVA results.

While a reciprocal transplant experiment in the field would be preferred, it was not possible to obtain permits to translocate corals among our sites, and so we used a common garden experimental design to examine source population (deme) by pH treatment (habitat) interactions to look for signals of local adaptation in home vs. away and local vs. foreign conditions (*sensu* Kawecki & Ebert^52^). To fulfill the home vs. away criterion we would expect higher mean performance in the ‘sympatric’ deme × habitat combination as compared to the ‘allopatric’ one (i.e., ramets should show higher performance under conditions approximating the home site than they do under conditions from elsewhere). To fulfill the local vs. foreign criterion the local demes should show higher mean performance in that habitat than the foreign ones (i.e., demes from low pH environments should experience higher mean performance under low pH than demes from high pH environments, and vice versa). We plot the source population (high or low pH as outlined above) against performance (measured as calcification rate) in both ambient and acidified conditions for each species to compare local vs. immigrant genotype responses to experimentally increased pCO_2_ (Fig. 4). Only *Montipora patula* shows a statistically significant crossed local vs. foreign slope (Fig. 4) suggestive of divergent selection for pH tolerance, but not necessarily local adaptation^52^. It is important to note that even for this single case of crossed slopes, there is no change in calcification rate among the corals from low pH sources rather than opposing reaction norms in home vs. away conditions. In every other species, both high and low pH source sites show statistically similar parallel slopes and generally increased rates of calcification under high pH conditions regardless of source population, which is inconsistent with either the home vs. away or the local vs. foreign criteria (Fig. 4). For three of these species (*Pocillopora meandrina, Montipora capitata*, and *M. flabellata*), calcification rates were significantly higher among individuals from low pH source sites, whereas for *Porites lobata*, calcification was significantly higher among individuals from high pH sites (Table S3).

### Species-specific acidification tolerances

Among the eight coral species examined, *Porites lobata* was significantly more sensitive to acidification than four of the species (*Pocillopora meandrina, Montipora capitata, Porites compressa*, and *P. evermanni*), whereas the remaining three species (*Pocillopora acuta, Montipora flabellata*, and *M. patula*) showed intermediate sensitivity to reduced pH (Fig. S2; Table S4).

## Discussion

There is considerable debate regarding predictions of coral reef responses to ocean acidification^1,19,23,26,53–56^. Although many have discounted adaptation as a realistic option for the future of coral reefs there is both circumstantial evidence for local adaptation of corals to regional environments^57–59^, and experimental evidence for divergent selection across surprisingly small spatial scales^20,21,34,60,61^, but the potential timescale of adaptation remains unknown. Such findings are important because even a relatively small degree of acclimatization and adaptation can dramatically alter future predictions for coral reefs under climate change^11,12^. Long-term, multigenerational studies that examine the potential for coral adaptation to changing climate are lacking, but such studies exist for some calcifying marine organisms. For example, Lohbeck *et al*.^62^ found that the calcifying coccolithophore *Emiliania huxleyi* adapted to higher pCO_2_ over 500 generations such that cells grown under these conditions showed up to 50% higher calcification rates than those raised under ambient pCO_2_. A subsequent study by Benner *et al*.^63^ also found that over the course of 700 generations, these coccolithophores showed adaptation to the combined stress of warmed acidified water, and could build their plated carbonate skeletons despite elevated pCO_2_ and temperature conditions. Other work indicates that higher pCO_2_ does not necessarily reduce calcification rates given the ability of organisms to exert biological control over calcification^10,28,64^. Together these studies show the biological capacity for at least some calcifying organisms to adapt to acidification, but we do not foresee a study on coral responses across hundreds of generations any time in the near future.

It is well known that corals vary in their thermal tolerances among geographic locations with differing climates, and some evidence exists for adaptation to such differences^34,61^, but it is often assumed that significant temperature adaptation requires millenia. In contrast to such assumptions, a recent experiment replicated a study from 1970 to determine bleaching and mortality threshold temperatures in Hawaiian corals and found these thresholds to have shifted up to 2.2 °C higher in ~50 years, leading the authors to conclude that corals had either acclimatized or adapted to higher ocean temperatures over decades as opposed to millenia^9,65–67^. Along similar lines, Kenkel and colleagues^34^ examined populations of the mustard hill coral, *Porites astreoides*, to evaluate whether increased thermal stress tolerance inshore was a function of long-term physiological acclimatization or genetic adaptation and found ~10–15% of variation in growth was attributable to heritable differences among recruits after maternal effects were taken into account. With the accumulation of such examples, factors such as long lifespan and broad dispersal, long assumed to preclude adaptation of corals to rapid climate change, are being questioned as it is becoming clear that corals are neither as universally long-lived nor as broadly dispersive as once thought^58,68–72^. Ultimately, however, the question of whether or not scleractinian corals are able to mount an adaptive response depends critically on whether or not responses to ocean acidification are variable and heritable, but such studies are currently lacking. As Kelly and Hofmann point out in their review^73^, there is precious little data on adaptation of marine organisms to ocean acidification; yet this missing information is critically important to predicting the capacity for selection to drive adaptation to future ocean conditions. Here, we present the first estimate of broad sense heritability of calcification rate in response to increased pCO_2_ for the eight most common coral species in Hawaiʻi to address this critical knowledge gap.

### Heritability of calcification rates

Calcification rates were significantly heritable across all eight species, within three diverse families of scleractinian corals. Further, the estimates of heritability (ranging from 0.32 to 0.61) were surprisingly high, considering that heritability of life-history traits that are connected to fitness tend to have much lower heritability than morphological traits^74,75^. Life-history heritability values are often low because most populations are expected to be near evolutionary optima with relatively low genetic variance contributing to total fitness^74^. Although we cannot ascribe the heritability directly to additive genetic variation (because we calculated only broad-sense – H^2^ – heritability as opposed to narrow-sense – *h*^2^) here, the high heritability values reported for these species nonetheless suggests that a large portion of the observed variation in calcification rates among unrelated coral colonies can be explained by heritable genetic factors. The high heritability values combined with considerable variation among individual colonies (Fig. 2) and very large population sizes^76^ indicates high potential for selection to act on calcification rates even if there is population genetic structure among the collection locations. Although we have only eight species from which to generalize, the corals included in our study represent three divergent families (Acroporidae, Pocilloporidae, and Poritidae), and both major evolutionary clades of scleractinian corals (Complexa and Robusta). The complex and robust clades of corals split ~420 million years ago, and within the clades, these coral families diverged >100 million years ago^77,78^. Thus, even among our limited taxonomic sampling of eight species here, the fact that every species of the several distinct lineages across the scleractinian tree of life all show high heritability suggests the trend is likely widespread, and that there is broad potential for selection to act on rates of calcification in response to ocean acidification.

### Individual variation

We find that overall mean calcification rate is decreased by experimental acidification in all eight species of Hawaiian scleractinians (Fig. S2), but that individual colonies within each species show highly variable responses relative to one another (Fig. 2). Looking across species, we see a general tendency that faster growing species are more impacted by acidification than are the more slowly growing ones, consistent with previous findings^54,79–81^. However, the substantial individual variability is hidden when looking at only mean calcification rates, which are all reduced under increased pCO_2_, and it is particularly noteworthy that at least one individual of each species tested maintained or even increased calcification rate under experimental acidification (Fig. 2).

The high variability among both species and conspecific individuals in response to altered pH, coupled with high heritability indicates more complexity in this relationship than previously appreciated. The fact that the source location predicts mean response to experimental manipulation of pH for only two of our eight species, and that we observe virtually every possible outcome from the eight species that we studied (Fig. 2), also implies that studies of single exemplar species are likely of little predictive power to be generalized to outcomes of future climate change scenarios. Further, the fact that for six of these eight species the individual variation in acidification tolerance within each site is roughly equal to the variation among sites suggests that increased sampling of genetic individuals is necessary for generalizations; both pH susceptible and robust individuals are distributed across populations, although not necessarily at equal frequencies (Figs. 3,4).

### Physiological acclimatization, local adaptation or something else?

Responses to experimentally reduced pH have been studied in a variety of coral species, but unlike temperature, there remains little evidence that variation in calcification rates among individuals can result from physiological acclimatization of adult coral colonies. Under experimental acidification, *Montipora capitata* and *Stylophora pistillata* failed to show any signs of acclimatization over timescales ranging from 24 hr to 1 yr^82,83^ nor did *Porites astreoides* show evidence of acclimatization when collected from naturally acidified field sites^84^. Although evidence of acclimatization to acidification is lacking, relatively few scleractinian species have been tested to date, and only one of the species in this study (*M.capitata*) are among those tested to date. Further, it is possible that the ability to acclimatize is limited to early in the life cycle (perhaps during metamorphosis from larva to coral polyp), or development within a given environment leads to canalization of the physiological responses of the organism for the rest of their life. However, if this early life history mechanism were true, we should expect that colonies collected from sites with differing environmental conditions around Oʻahu should show more similar responses than colonies from different pH environments, which is not supported by these data. Likewise, to the best of our knowledge, only one study to date has examined the effects of larval pH environment on coral performance. Indeed, larvae of *P. acuta* showed altered respiratory responses if their maternal colony was held under acidified and warmed conditions while they were being brooded^85^, but those experiments did not follow individuals further to determine whether these differences last beyond metamorphosis in the adult colony. Our data suggest that this increased acidification tolerance via larval preconditioning is unlikely permanent, because we find no significant difference between the mean calcification rates of adult *P. acuta* collected from naturally high or low pH sites around Oʻahu (Fig. 4).

Likewise, when we plot the source population by habitat interaction for each species, we find none that clearly satisfy both the classic ‘local vs. foreign’ and ‘home vs. away’ criteria^52,86^ for local adaptation (Fig. 4). In fact, we find almost all possible outcomes with these eight species, ranging from nearly identical responses among treatments regardless of the colony source (*Pocillopora acuta*, *Porites compressa, P. evermanni*), to consistently higher growth of colonies from low pH sources (*P. meandrina*, *M. capitata*, *M. flabellata*), to consistently higher growth of colonies from high pH sources (*P. lobata*). It is particularly noteworthy that the only species, *M. patula* that shows crossed responses in the local vs. foreign treatments fails to meet the home vs. away criterion (Fig. 4). This pattern is consistent with divergent selection but not necessarily local adaptation.

There are four primary evolutionary stable solutions for a sedentary organism with dispersal among variable habitats such as reef corals in a marine metapopulation :^52,87^ (1) plasticity that allows the organism to match the habitat within which they settle (i.e., acclimatization); (2) local adaptation among habitats; (3) adaptation to the mean (or most common) habitat; or (4) a protected polymorphism, in which each type is at an advantage in specific habitats and is therefore protected from extinction^88,89^. If the corals had acclimatized to the common garden environment or were adapted to the mean environment, then we should have found little individual variation in response to experimental acidification and low heritability estimates. Instead we find substantial individual variation within treatments and high heritability of calcification rates, contrary to the predictions of these mechanisms. It is important to note, however, that we cannot exclude the possibility of developmental plasticity and canalization of performance as an explanation for the significant site effects observed with *M. patula* and *P. meandrina*. To evaluate which of these four strategies is most likely, we need to consider the competing evidence for all alternatives. If the corals were locally adapted then we should have seen crossing reaction norms in local vs. foreign treatments, yet we only observe this pattern for a single species (*M. patula*) in our study (Fig. 4). Even for this species, we see no evidence of reduced performance when individuals from a low pH environment are held under away conditions, making local adaptation a less likely explanation^52,86,90^. When the spatial scale of divergent selection is smaller than that of gene flow, a protected polymorphism is more likely to emerge than local adaptation^52,91^. Indeed, natural mosaics in both temperature and pH between Kāneʻohe Bay and adjacent coastal waters provide just such an opportunity^10,30,92^. In our study area, seawater pH varies markedly in magnitude and variability over a scale of <20 km^30,92^, which should be within easy dispersal distances for these corals^39–41^. In addition, possible gradients among depths as well as between onshore-offshore comparisons also provide scope for habitats in which differences in pH and temperature tolerance may be favored^20,34,60,61^. Hence, the data we present here are most consistent with a protected polymorphism in which variable tolerance to pH is maintained in the population because it is favored differentially among spatially or temporally variable habitats.

Our finding here is even more striking because we know that sewage discharge nearly extirpated corals from Kāneʻohe Bay by the mid-1970s^93,94^, but following diversion of the sewage outfalls in 1978, corals rapidly recovered to reach among the highest coral cover in the State^12,31,32^. Studies of thermal tolerance performed following the same experimental protocols through time have shown that some of these corals have increased their mean thermal tolerance by up to 2.2 °C in the intervening decades^9,65,66^. Given considerable individual variation in calcification rates, high heritability, and the recovery of coral cover within the naturally warmer and more acidic portion of Kāneʻohe Bay within about 30 years^31^, accumulating data suggest that corals may be capable of mounting an adaptive response to anthropogenic climate change much more rapidly than previously thought possible.

### Summary and conclusions

Here, we examined inter-individual variation and heritability of calcification rate among eight of the dominant Hawaiian coral species under both ambient and low pH conditions, similar to those predicted after a century of high CO_2_ emissions^95^. We show that coral calcification rates varied significantly among species, conspecific colonies and pH treatments, with significant interactions between colony and pH treatment in only 2 of 8 species (*Pocillopora meandrina* and *Porites evermanni*). Likewise, there are significant interactions between site and pH treatment in only 2 species (*Montipora patula* and *Pocillopora meandrina*). Broad sense heritability of calcification rates was high among all eight species, ranging from 0.32 in *Porites evermanni* to 0.61 in *Porites compressa*.

The high heritability and considerable variation of pH tolerances among individuals implies that selection can act on scleractinian calcification rates in nature and that populations could adapt to changes in ocean chemistry with sufficient time. Given the history of Kāneʻohe Bay and the data presented here, the scope for adaptation apparently exists and selection in response to ocean acidification may be more rapid than previously thought for corals. Despite this study being an important first step, we caution that ocean acidification is not the only physical factor controlling coral calcification, and that growth rate alone is a poor predictor of fitness^96^. Temperature, irradiance, flow, and nutrient concentrations all play significant roles in the growth rate of corals among geographic locations^97–103^, and we know very little about either synergies or trade-offs among responses to acidification and these other factors in controlling coral fitness. Future studies that incorporate multifactorial stressors, and consider interactions and trade-offs among them, should be performed to better predict how scleractinian corals might respond under global change scenarios.

Our study indicates a strong interaction of pH × Site and pH × Colony in which some individuals are more resistant to acidification than others, and in fact, at least one individual of every species tested calcified as fast or faster under acidification than at ambient pH. In contrast to predictions of local adaptation, however, for most of the species these individuals are distributed apparently at random with respect to the physical environments sampled, and the lack of home vs. away trade-offs suggests a protected polymorphism (as opposed to local adaptation) is the most likely strategy. A better understanding of the scope and distribution of this individual variation among genetic individuals is critical to understanding and predicting responses of reefs to future conditions. Further, such extreme variation among individuals highlights our lack of a mechanistic understanding of how acidification affects calcification in corals, because none of the current models of coral calcification predict such individual variability^17,82^. Regardless of the mechanism, the consistently high heritability values reported for all eight species tested here provides evidence that sensitivity to acidification is a trait under heritable genetic control, and that there is considerable variation among both species and individuals on which selection may act. While we tested only eight species of corals here, they represent highly divergent lineages that include the most important families of reef building corals globally, implying that such heritable differences in response to acidification are likely widespread in scleractinians. Such individual variation is rarely considered explicitly in coral studies and may help to explain conflicting results among some studies. Therefore, genetic identity of individuals should be considered explicitly in studies trying to generalize experimental results to population responses. The most important implication of our study is that selection is likely already acting on pH tolerance in many scleractinians, which if the response is quick enough, in turn provides a more positive outlook for coral reefs under future global change, particularly with reduced CO_2_ emissions.

## Supplementary information

Supplementary Information 

## Data Availability

Data are archived in the public repository Figshare at 10.6084/m9.figshare.11421297.v1.
