# Supplementary material for: High heritability of coral calcification rates and evolutionary potential under ocean acidification
Source: Sci Rep. 2019 Dec 31;9:20419. doi: 10.1038/s41598-019-56313-1 (PMC6938506; doi:10.1038/s41598-019-56313-1)
Supplement: Supplementary file 1 — Supplementary Information [file 41598_2019_56313_MOESM1_ESM.docx]

**Supplementary information for: “High heritability of coral calcification rates and evolutionary potential under ocean acidification”**

**Authors: Christopher P. Jury, Mia N. Delano, and Robert J. Toonen**

**Table S1.** ANOVA results for treatment effects of pH, collection site, and coral colony on calcification rates for each species. Fits are provided both with and without the nubbins which died included in the model (see text for details). Note: for *P. compresssa* there was no mortality so contrasts with and without dead nubbins included are identical. df = degrees of freedom; SS = sum of squares; MS = mean square; F = variation between sample means / variation within the samples. P-values in bold are significant at α= 0.05.

|  | df | SS | MS | F | p |
| --- | --- | --- | --- | --- | --- |
| *P. acuta* |  |  |  |  |  |
| Without dead |  |  |  |  |  |
| pH | 1 | 20.651 | 20.651 | 26.421 | **<0.001** |
| Site | 2 | 2.411 | 1.206 | 1.542 | 0.23 |
| Colony | 5 | 30.943 | 6.189 | 7.918 | **<0.001** |
| pH×Site | 2 | 0.550 | 0.275 | 0.352 | 0.71 |
| pH×Colony | 5 | 2.593 | 0.519 | 0.663 | 0.65 |
| Residuals | 29 | 22.667 | 0.782 |  |  |
|  |  |  |  |  |  |
| With dead |  |  |  |  |  |
| pH | 1 | 13.16 | 13.164 | 17.682 | **<0.001** |
| Site | 2 | 0.85 | 0.426 | 0.573 | 0.57 |
| Colony | 5 | 45.20 | 9.040 | 12.143 | **<0.001** |
| pH×Site | 2 | 0.92 | 0.461 | 0.619 | 0.54 |
| pH×Colony | 5 | 4.56 | 0.912 | 1.225 | 0.32 |
| Residuals | 32 | 23.82 | 0.744 |  |  |
|  |  |  |  |  |  |
| *P. meandrina* |  |  |  |  |  |
| Without dead |  |  |  |  |  |
| pH | 1 | 15.46 | 15.455 | 38.544 | **<0.001** |
| Site | 3 | 54.63 | 18.209 | 45.410 | **<0.001** |
| Colony | 11 | 45.41 | 4.129 | 10.296 | **<0.001** |
| pH×Site | 3 | 4.27 | 1.425 | 3.554 | **0.02** |
| pH×Colony | 11 | 9.78 | 0.889 | 2.217 | **0.03** |
| Residuals | 56 | 22.46 | 0.401 |  |  |
|  |  |  |  |  |  |
| With dead |  |  |  |  |  |
| pH | 1 | 18.61 | 18.606 | 33.346 | **<0.001** |
| Site | 3 | 53.01 | 17.670 | 31.668 | **<0.001** |
| Colony | 11 | 50.51 | 4.592 | 8.229 | **<0.001** |
| pH×Site | 3 | 3.63 | 1.210 | 2.168 | 0.10 |
| pH×Colony | 11 | 11.89 | 1.081 | 1.937 | 0.05 |
| Residuals | 60 | 33.48 | 0.558 |  |  |
|  |  |  |  |  |  |
| *M. capitata* |  |  |  |  |  |
| Without dead |  |  |  |  |  |
| pH | 1 | 20.93 | 20.927 | 16.869 | **<0.001** |
| Site | 4 | 56.31 | 14.078 | 11.348 | **<0.001** |
| Colony | 10 | 91.72 | 9.172 | 7.393 | **<0.001** |
| pH×Site | 4 | 3.61 | 0.903 | 0.728 | 0.58 |
| pH×Colony | 10 | 12.19 | 1.219 | 0.982 | 0.47 |
| Residuals | 59 | 73.19 | 1.241 |  |  |
|  |  |  |  |  |  |
| With dead |  |  |  |  |  |
| pH | 1 | 23.42 | 23.418 | 18.744 | **<0.001** |
| Site | 4 | 59.45 | 14.863 | 11.896 | **<0.001** |
| Colony | 10 | 91.97 | 9.197 | 7.361 | **<0.001** |
| pH×Site | 4 | 3.38 | 0.845 | 0.676 | 0.61 |
| pH×Colony | 10 | 12.27 | 1.227 | 0.982 | 0.47 |
| Residuals | 60 | 74.96 | 1.249 |  |  |
|  |  |  |  |  |  |
| *M. flabellata* |  |  |  |  |  |
| Without dead |  |  |  |  |  |
| pH | 1 | 26.52 | 26.52 | 8.130 | **<0.01** |
| Site | 1 | 35.65 | 35.65 | 10.932 | **<0.01** |
| Colony | 6 | 117.60 | 19.60 | 6.010 | **<0.001** |
| pH×Site | 1 | 1.32 | 1.32 | 0.406 | 0.53 |
| pH×Colony | 6 | 30.46 | 5.08 | 1.557 | 0.19 |
| Residuals | 31 | 101.10 | 3.261 |  |  |
|  |  |  |  |  |  |
| With dead |  |  |  |  |  |
| pH | 1 | 32.31 | 32.31 | 9.605 | **<0.01** |
| Site | 1 | 32.18 | 32.18 | 9.567 | **<0.01** |
| Colony | 6 | 129.23 | 21.54 | 6.404 | **<0.001** |
| pH×Site | 1 | 1.66 | 1.66 | 0.494 | 0.49 |
| pH×Colony | 6 | 25.99 | 4.33 | 1.288 | 0.29 |
| Residuals | 32 | 107.63 | 3.36 |  |  |
|  |  |  |  |  |  |
| *M. patula* |  |  |  |  |  |
| Without dead |  |  |  |  |  |
| pH | 1 | 32.34 | 32.34 | 54.464 | **<0.001** |
| Site | 3 | 10.82 | 3.61 | 6.072 | **<0.01** |
| Colony | 11 | 36.58 | 3.33 | 5.600 | **<0.001** |
| pH×Site | 3 | 10.40 | 3.47 | 5.838 | **<0.01** |
| pH×Colony | 11 | 11.04 | 1.00 | 1.689 | 0.10 |
| Residuals | 58 | 34.44 | 0.59 |  |  |
|  |  |  |  |  |  |
| With dead |  |  |  |  |  |
| pH | 1 | 31.63 | 31.63 | 44.781 | **<0.001** |
| Site | 3 | 7.13 | 2.38 | 3.364 | **0.02** |
| Colony | 11 | 43.44 | 3.95 | 5.592 | **<0.001** |
| pH×Site | 3 | 9.27 | 3.09 | 4.373 | **<0.01** |
| pH×Colony | 11 | 10.55 | 0.96 | 1.358 | 0.22 |
| Residuals | 60 | 42.37 | 0.71 |  |  |
|  |  |  |  |  |  |
| *P. compressa* |  |  |  |  |  |
| pH | 1 | 18.94 | 18.942 | 16.865 | **<0.001** |
| Site | 4 | 36.64 | 9.161 | 8.157 | **<0.001** |
| Colony | 10 | 111.71 | 11.171 | 9.946 | **<0.001** |
| pH×Site | 4 | 4.93 | 1.232 | 1.097 | 0.37 |
| pH×Colony | 10 | 6.82 | 0.682 | 0.607 | 0.80 |
| Residuals | 60 | 67.39 | 1.123 |  |  |
|  |  |  |  |  |  |
| *P. evermanni* |  |  |  |  |  |
| Without dead |  |  |  |  |  |
| pH | 1 | 13.26 | 13.261 | 16.760 | **<0.001** |
| Site | 4 | 13.96 | 3.491 | 4.412 | **<0.01** |
| Colony | 10 | 33.76 | 3.376 | 4.267 | **<0.001** |
| pH×Site | 4 | 3.44 | 0.859 | 1.086 | 0.37 |
| pH×Colony | 10 | 18.71 | 1.871 | 2.364 | **0.02** |
| Residuals | 52 | 41.14 | 0.791 |  |  |
|  |  |  |  |  |  |
| With dead |  |  |  |  |  |
| pH | 1 | 25.61 | 25.612 | 27.027 | **<0.001** |
| Site | 4 | 15.39 | 3.846 | 4.059 | **<0.01** |
| Colony | 10 | 37.51 | 3.751 | 3.958 | **<0.001** |
| pH×Site | 4 | 4.48 | 1.121 | 1.182 | 0.33 |
| pH×Colony | 10 | 30.74 | 3.074 | 3.243 | **<0.01** |
| Residuals | 60 | 56.86 | 0.948 |  |  |
|  |  |  |  |  |  |
| *P. lobata* |  |  |  |  |  |
| Without dead |  |  |  |  |  |
| pH | 1 | 90.32 | 90.32 | 76.311 | **<0.001** |
| Site | 4 | 40.32 | 10.08 | 8.516 | **<0.001** |
| Colony | 10 | 62.20 | 6.22 | 5.255 | **<0.001** |
| pH×Site | 4 | 3.98 | 1.00 | 0.841 | 0.50 |
| pH×Colony | 10 | 22.48 | 2.25 | 1.899 | 0.06 |
| Residuals | 59 | 69.83 | 1.18 |  |  |
|  |  |  |  |  |  |
| With dead |  |  |  |  |  |
| pH | 1 | 96.74 | 96.74 | 72.167 | **<0.001** |
| Site | 4 | 40.82 | 10.21 | 7.613 | **<0.001** |
| Colony | 10 | 57.59 | 5.76 | 4.296 | **<0.001** |
| pH×Site | 4 | 4.47 | 1.12 | 0.834 | 0.51 |
| pH×Colony | 10 | 22.30 | 2.23 | 1.663 | 0.11 |
| Residuals | 60 | 80.43 | 1.34 |  |  |

**Table S2.** Broad-sense heritability **(**H^2^) values and the 95% confidence interval for the estimate of heritability of calcification rate for each of the eight Hawaiian coral species tested when different combinations of covariates are included in the model as well as with and without the dead nubbins included in the analyses. Regardless of the covariates or dead nubbins included, none of the heritability estimates differ significantly for any of the species. Heritability estimates are shown as unbracketed values and 95% CIs are shown in parentheses.

| Covariates included | | | | |
| --- | --- | --- | --- | --- |
|  | **None** | **pH** | **Site** | **pH+Site** |
| Without dead |  |  |  |  |
| *P. acuta* | 0.40 (0.12-0.78) | 0.50 (0.21-0.84) | 0.49 (0.20-0.83) | 0.58 (0.29-0.87) |
| *P. meandrina* | 0.58 (0.37-0.80) | 0.69 (0.50-0.86) | 0.42 (0.21-0.68) | 0.55 (0.33-0.77) |
| *M. capitata* | 0.50 (0.29-0.74) | 0.56 (0.35-0.78) | 0.46 (0.25-0.72) | 0.52 (0.31-0.76) |
| *M. patula* | 0.24 (0.06-0.52) | 0.36 (0.16-0.64) | 0.23 (0.06-0.51) | 0.36 (0.16-0.63) |
| *M. flabellata* | 0.42 (0.14-0.78) | 0.47 (0.19-0.82) | 0.38 (0.11-0.76) | 0.44 (0.16-0.80) |
| *P. compressa* | 0.54 (0.33-0.77) | 0.60 (0.39-0.80) | 0.56 (0.35-0.78) | 0.61 (0.41-0.81) |
| *P. evermanni* | 0.27 (0.08-0.56) | 0.32 (0.12-0.60) | 0.27 (0.08-0.55) | 0.32 (0.11-0.60) |
| *P. lobata* | 0.25 (0.07-0.53) | 0.43 (0.22-0.69) | 0.21 (0.04-0.49) | 0.39 (0.18-0.65) |
|  |  |  |  |  |
| With dead |  |  |  |  |
| *P. acuta* | 0.46 (0.18-0.81) | 0.56 (0.28-0.86) | 0.56 (0.27-0.86) | 0.65 (0.37-0.89) |
| *P. meandrina* | 0.55 (0.33-0.77) | 0.63 (0.43-0.82) | 0.41 (0.20-0.67) | 0.50 (0.28-0.74) |
| *M. capitata* | 0.50 (0.29-0.74) | 0.57 (0.36-0.78) | 0.46 (0.25-0.71) | 0.52 (0.31-0.75) |
| *M. patula* | 0.24 (0.06-0.52) | 0.35 (0.15-0.63) | 0.26 (0.08-0.54) | 0.38 (0.18-0.65) |
| *M. flabellata* | 0.43 (0.15-0.79) | 0.48 (0.20-0.82) | 0.41 (0.14-0.78) | 0.46 (0.18-0.81) |
| *P. compressa* | 0.54 (0.33-0.77) | 0.60 (0.39-0.80) | 0.56 (0.35-0.78) | 0.61 (0.41-0.81) |
| *P. evermanni* | 0.19 (0.03-0.47) | 0.25 (0.07-0.53) | 0.19 (0.03-0.46) | 0.25 (0.07-0.53) |
| *P. lobata* | 0.21 (0.04-0.49) | 0.39 (0.19-0.66) | 0.16 (0.01-0.43) | 0.33 (0.14-0.61) |

**Table S3.** ANOVA results for treatment effects of pH, pH of origin, and collection site on calcification rates for each species. Fits are provided both with and without the nubbins which died included in the model (see text for details). Note: for *M. flabellata* contrasts by pH of origin and collection site are equivalent because this species was collected at two sites; for *P. compresssa* there was no mortality so contrasts with and without dead nubbins included are the same. df = degrees of freedom; SS = sum of squares; MS = mean square; F = variation between sample means / variation within the samples. P-values in bold are significant at α= 0.05.

|  | df | SS | MS | F | p |
| --- | --- | --- | --- | --- | --- |
| *P. acuta* |  |  |  |  |  |
| Without dead |  |  |  |  |  |
| pH | 1 | 20.65 | 20.651 | 14.519 | **<0.001** |
| pH.origin | 1 | 0.01 | 0.012 | 0.008 | 0.93 |
| Site | 1 | 2.40 | 2.400 | 1.687 | 0.20 |
| pH×pH.origin | 1 | 0.8 | 0.081 | 0.057 | 0.81 |
| pH×Site | 1 | 1.20 | 1.198 | 0.842 | 0.36 |
| Residuals | 39 | 55.47 | 1.422 |  |  |
|  |  |  |  |  |  |
| With dead |  |  |  |  |  |
| pH | 1 | 13.16 | 13.164 | 7.514 | **<0.001** |
| pH.origin | 1 | 0.53 | 0.529 | 0.302 | 0.59 |
| Site | 1 | 0.32 | 0.323 | 0.185 | 0.67 |
| pH×pH.origin | 1 | 0.92 | 0.916 | 0.523 | 0.470.95 |
| pH×Site | 1 | 0.01 | 0.006 | 0.003 |  |
| Residuals | 42 | 73.58 | 1.752 |  |  |
|  |  |  |  |  |  |
| *P. meandrina* |  |  |  |  |  |
| Without dead |  |  |  |  |  |
| pH | 1 | 15.46 | 15.455 | 15.770 | **<0.001** |
| pH.origin | 1 | 26.45 | 26.455 | 26.993 | **<0.001** |
| Site | 2 | 28.17 | 14.085 | 14.372 | **<0.001** |
| pH×pH.origin | 1 | 1.72 | 1.724 | 1.759 | 0.19 |
| pH×Site | 2 | 3.75 | 1.877 | 1.915 | 0.15 |
| Residuals | 78 | 76.44 | 0.980 |  |  |
|  |  |  |  |  |  |
| With dead | 1 | 18.61 | 18.606 | 15.913 | **<0.001** |
| pH | 1 | 30.67 | 30.668 | 26.228 | **<0.001** |
| pH.origin | 2 | 22.34 | 11.171 | 9.554 | **<0.001** |
| Site | 1 | 1.53 | 1.534 | 1.312 | 0.26 |
| pH×pH.origin | 2 | 2.10 | 1.048 | 0.896 | 0.41 |
| pH×Site | 82 | 95.88 | 1.169 |  |  |
| Residuals |  |  |  |  |  |
|  |  |  |  |  |  |
| *M. capitata* |  |  |  |  |  |
| Without dead |  |  |  |  |  |
| pH | 1 | 20.93 | 20.93 | 9.935 | **<0.01** |
| pH.origin | 1 | 55.25 | 55.25 | 26.645 | **<0.001** |
| Site | 3 | 1.06 | 0.35 | 0.158 | 0.92 |
| pH×pH.origin | 1 | 2.65 | 2.65 | 1.183 | 0.28 |
| pH×Site | 3 | 0.96 | 0.32 | 0.143 | 0.93 |
| Residuals | 79 | 177.10 | 2.24 |  |  |
|  |  |  |  |  |  |
| With dead |  |  |  |  |  |
| pH | 1 | 23.42 | 23.42 | 10.454 | **<0.01** |
| pH.origin | 1 | 57.76 | 57.76 | 25.782 | **<0.001** |
| Site | 3 | 1.70 | 0.57 | 0.253 | 0.86 |
| pH×pH.origin | 1 | 2.25 | 2.25 | 1.004 | 0.32 |
| pH×Site | 3 | 1.13 | 0.38 | 0.168 | 0.92 |
| Residuals | 80 | 179.21 | 2.24 |  |  |
|  |  |  |  |  |  |
| *M. flabellata* |  |  |  |  |  |
| Without dead |  |  |  |  |  |
| pH | 1 | 26.52 | 26.52 | 4.569 | **0.04** |
| pH.origin | 1 | 35.65 | 35.65 | 6.143 | **0.02** |
| pH×pH.origin | 1 | 0.95 | 0.95 | 0.163 | 0.69 |
| Residuals | 43 | 249.55 | 5.80 |  |  |
|  |  |  |  |  |  |
| With dead |  |  |  |  |  |
| pH | 1 | 32.31 | 32.31 | 5.408 | **0.02** |
| pH.origin | 1 | 32.18 | 32.18 | 5.387 | **0.03** |
| pH×pH.origin | 1 | 1.66 | 1.66 | 0.278 | 0.60 |
| Residuals | 44 | 262.86 | 5.97 |  |  |
|  |  |  |  |  |  |
| *M. patula* |  |  |  |  |  |
| Without dead |  |  |  |  |  |
| pH | 1 | 32.34 | 32.34 | 31.531 | **<0.001** |
| pH.origin | 1 | 0.36 | 0.36 | 0.351 | 0.55 |
| Site | 2 | 10.46 | 5.23 | 5.097 | **<0.01** |
| pH×pH.origin | 1 | 8.82 | 8.82 | 8.598 | **<0.01** |
| pH×Site | 2 | 1.58 | 0.79 | 0.771 | 0.47 |
| Residuals | 80 | 82.06 | 1.03 |  |  |
|  |  |  |  |  |  |
| With dead |  |  |  |  |  |
| pH | 1 | 31.63 | 31.63 | 26.911 | **<0.001** |
| pH.origin | 1 | 0.11 | 0.11 | 0.092 | 0.76 |
| Site | 2 | 7.02 | 3.51 | 2.986 | 0.06 |
| pH×pH.origin | 1 | 8.30 | 8.30 | 7.060 | **<0.01** |
| pH×Site | 2 | 0.97 | 0.48 | 0.412 | 0.66 |
| Residuals | 82 | 96.37 | 1.18 |  |  |
|  |  |  |  |  |  |
| *P. compressa* |  |  |  |  |  |
| pH | 1 | 18.94 | 18.942 | 8.150 | **<0.01** |
| pH.origin | 1 | 3.74 | 3.742 | 1.610 | 0.21 |
| Site | 3 | 32.90 | 10.968 | 4.719 | **<0.01** |
| pH×pH.origin | 1 | 0.19 | 0.193 | 0.083 | 0.77 |
| pH×Site | 3 | 4.74 | 1.579 | 0.679 | 0.57 |
| Residuals | 80 | 185.92 | 2.324 |  |  |
|  |  |  |  |  |  |
| *P. evermanni* |  |  |  |  |  |
| Without dead |  |  |  |  |  |
| pH | 1 | 13.26 | 13.261 | 10.246 | **<0.01** |
| pH.origin | 1 | 3.98 | 3.983 | 3.078 | 0.08 |
| Site | 3 | 9.98 | 3.327 | 2.571 | 0.06 |
| pH×pH.origin | 1 | 0.20 | 0.205 | 0.158 | 0.69 |
| pH×Site | 3 | 3.66 | 1.220 | 0.943 | 0.42 |
| Residuals | 72 | 93.18 | 1.294 |  |  |
|  |  |  |  |  |  |
| With dead |  |  |  |  |  |
| pH | 1 | 25.61 | 25.612 | 16.378 | **<0.001** |
| pH.origin | 1 | 5.18 | 5.181 | 3.313 | 0.07 |
| Site | 3 | 10.20 | 3.402 | 2.175 | 0.10 |
| pH×pH.origin | 1 | 0.20 | 0.195 | 0.125 | 0.72 |
| pH×Site | 3 | 4.29 | 1.429 | 0.914 | 0.44 |
| Residuals | 80 | 125.10 | 1.564 |  |  |
|  |  |  |  |  |  |
| *P. lobata* |  |  |  |  |  |
| Without dead |  |  |  |  |  |
| pH | 1 | 90.32 | 90.32 | 46.187 | **<0.001** |
| pH.origin | 1 | 20.48 | 20.48 | 10.472 | **<0.01** |
| Site | 3 | 19.84 | 6.61 | 3.382 | **0.02** |
| pH×pH.origin | 1 | 0.75 | 0.75 | 0.382 | 0.54 |
| pH×Site | 3 | 3.26 | 1.09 | 0.555 | 0.65 |
| Residuals | 79 | 154.49 | 1.96 |  |  |
|  |  |  |  |  |  |
| With dead |  |  |  |  |  |
| pH | 1 | 96.74 | 96.74 | 48.723 | **<0.001** |
| pH.origin | 1 | 19.18 | 19.18 | 9.572 | **<0.01** |
| Site | 3 | 21.64 | 7.21 | 3.599 | **0.02** |
| pH×pH.origin | 1 | 0.53 | 0.53 | 0.263 | 0.61 |
| pH×Site | 3 | 3.94 | 1.31 | 0.656 | 0.58 |
| Residuals | 80 | 160.32 | 2.00 |  |  |

**Table S4.** ANCOVA results for pairwise contrasts of slopes of calcification rate as a function of treatment pH derived from LMM fits for each species. Table shows p-values by contrast; values in bold are significant at α = 0.05. These contrasts indicate that *P. lobata* was significantly more sensitive to reduced pH than four of the species (*P. meandrina, M. capitata, P. compressa,* and *P. evermanni*) whereas the remaining three species (*P. acuta, M. flabellata,* and *M. patula*) show intermediate sensitivity to acidification.

|  | *P.*  *acuta* | *P. meandrina* | *M. capitata* | *M. flabellata* | *M.*  *patula* | *P. compressa* | *P. evermanni* |
| --- | --- | --- | --- | --- | --- | --- | --- |
| *P. meandrina* | 0.99 |  |  |  |  |  |  |
| *M. capitata* | 1 | 1 |  |  |  |  |  |
| *M. flabellata* | 1 | 0.70 | 0.81 |  |  |  |  |
| *M. patula* | 1 | 0.98 | 1 | 0.99 |  |  |  |
| *P. compressa* | 0.99 | 1 | 1 | 0.72 | 0.99 |  |  |
| *P. evermanni* | 0.95 | 1 | 1 | 0.51 | 0.91 | 1 |  |
| *P. lobata* | 0.54 | **0.02** | **0.04** | 0.95 | 0.23 | **0.02** | **<0.01** |


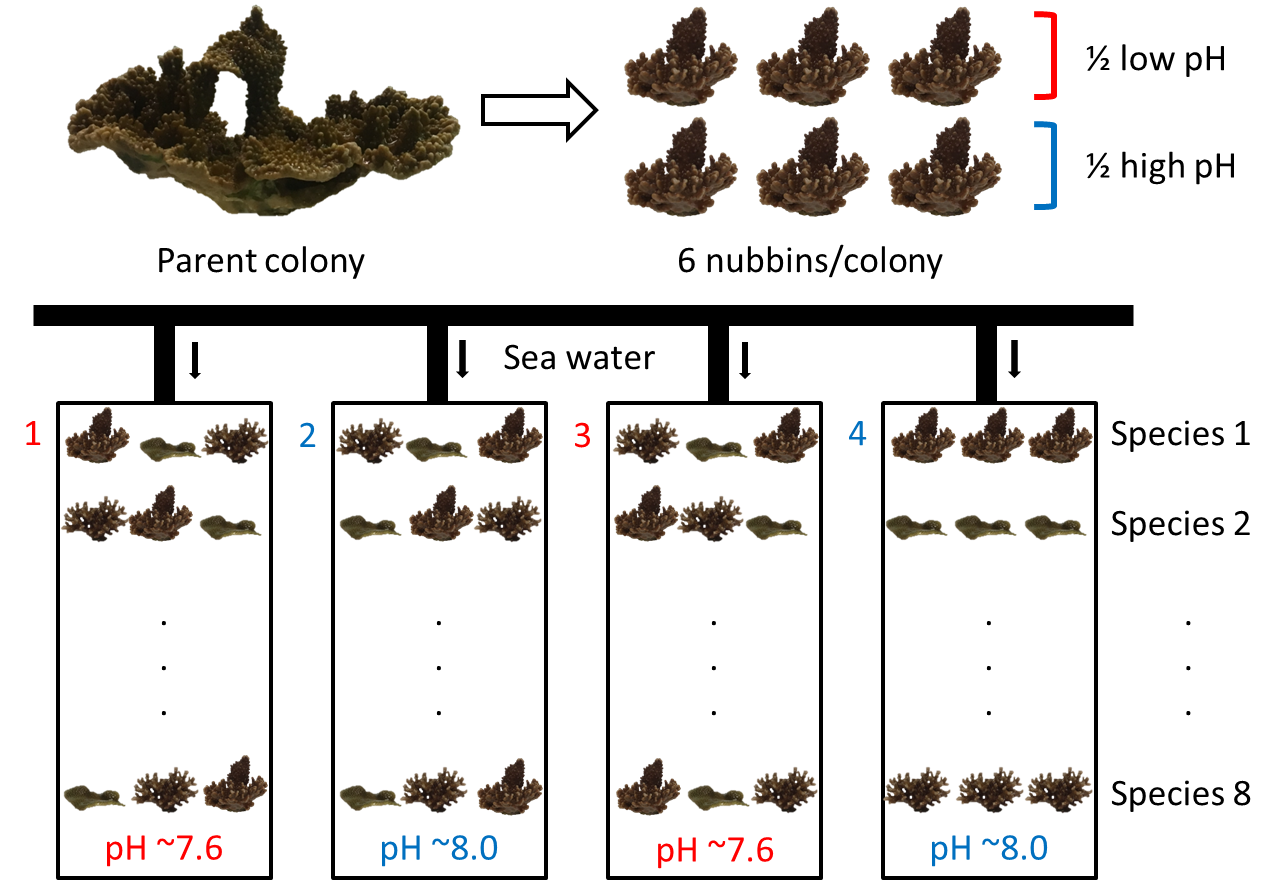


**Figure S1**. Experimental design for common garden heritability experiment. At least eight parent colonies of each of the eight species (*Pocillopora acuta*, *P. meandrina*, *Montipora capitata*, *M. patula*, *M. flabellata*, *Porites compressa*, *P. evermanni*, and *P. lobata*) from sites around Oʻahu (Table 2) were fragmented into six 3-5cm clonal ramets, attached to labeled plaster plugs, and allowed to recover (illustrated in Tank 4 above) prior to being assigned at random to treatment tanks (illustrated in Tanks 1-3 above). Clonal nubbins (ramets) from each parent colony (genets) were randomly assigned to pH treatments with 3 in the high (pH ~8.0) and 3 in the low (pH ~7.6) treatment flow-through mesocosm tanks (300L, 160 nubbins per tank). Flow-through conditions are explained in the text of the manuscript. Photos by C. P. Jury.

**Figure S2.** Mean calcification rate of coral nubbins of each of the eight primary reef building coral species in the Hawaiian Archipelago when held under either high or reduced pH conditions in the lab (error bars omitted for clarity). See Table S4 for ANCOVA results of contrasts among species.
